# Emicizumab prophylaxis in people with hemophilia A and inhibitors: a systematic review and meta-analysis

**DOI:** 10.1590/1516-3180.2023.0102.R1.20022024

**Published:** 2024-05-10

**Authors:** Tiago Paiva Prudente, Ricardo Mesquita Camelo, Rafael Alves Guimarães, Maria do Rosário Ferraz Roberti

**Affiliations:** I Medical student, Faculty of Medicine, Universidade Federal de Goiás (UFG), Goiânia (GO), Brazil.; II MD, PhD. Physician, Postdoctoral associate, Faculty of Medicine, Universidade Federal de Minas Gerais (UFMG), Belo Horizonte (MG), Brazil.; III RN, PhD. Epidemiologist, Professor, Faculty of Nursing, Universidade Federal de Goiás (UFG), Goiânia (GO), Brazil.; IV MD, PhD. Physician, Professor, Faculty of Medicine, Universidade Federal de Goiás (UFG), Goiânia (GO), Brazil; Physician, Secretaria de Saúde do Estado de Goiás (SES/GERAT), Goiânia (GO), Brazil.

**Keywords:** Hemophilia A, Antibodies, monoclonal, humanized, Blood coagulation factors, Emicizumab, Hemophilia A, Inhibitors, Prophylaxis, Annualized bleeding rate, Bypassing agents

## Abstract

**BACKGROUND:**

Until recently, the treatment of people with hemophilia A and inhibitors (PwHAi) was based on the use of bypassing agents (BPA). However, the advent of emicizumab as prophylaxis has demonstrated promising results.

**OBJECTIVES:**

We aimed to compare the bleeding endpoints between PwHAi on BPA and those on emicizumab prophylaxis.

**DESIGN AND SETTING:**

Systematic review of interventions and meta-analysis conducted at the Universidade Federal de Goiás, Goiânia, Goiás, Brazil.

**METHODS:**

The CENTRAL, MEDLINE, Scopus, and LILACS databases were searched on February 21, 2023. Two authors conducted the literature search, publication selection, and data extraction. The selected publications evaluated the bleeding endpoints between PwHAi on emicizumab prophylaxis and those on BPA prophylaxis. The risk of bias was evaluated according to the Joanna Briggs Institute criteria. A meta-analysis was performed to determine the annualized bleeding rate (ABR) for treated bleeds.

**RESULTS:**

Five publications (56 PwHAi) were selected from the 543 retrieved records. Overall, bleeding endpoints were lower during emicizumab prophylaxis than during BPA prophylaxis. All the publications had at least one risk of bias. The only common parameter for the meta-analysis was the ABR for treated bleeds. During emicizumab prophylaxis, the ABR for treated bleeds was lower than during BPA prophylaxis (standard mean difference: −1.58; 95% confidence interval −2.50, −0.66, P = 0.0008; *I* ^2^ = 68.4%, P = 0.0031).

**CONCLUSION:**

Emicizumab was superior to BPA in bleeding prophylaxis in PwHAi. However, both the small population size and potential risk of bias should be considered when evaluating these results.

**SYSTEMATIC REVIEW REGISTRATION:**

CRD42021278726, https://www.crd.york.ac.uk/prospero/display_record.php?RecordID=278726.

## INTRODUCTION

Hemophilia A (HA) is an X-linked recessive bleeding disorder characterized by reduced or absent coagulation factor (F) VIII activity.^1^ The clinical presentation depends on residual FVIII activity. Mild HA (FVIII 5%–40%) is characterized by increased bleeds mainly during surgery or after trauma, whereas severe HA (FVIII < 1%) is characterized by both spontaneous and provoked bleeds.^1^ Moderate HA (1%–5%) has a wide phenotype, depending on the residual FVIII activity.^1^ Bleeds occur mainly in joints, although they may also occur in vital organs.^1^ Consequently, joint bleeds lead to arthropathy and worsen quality of life.^1^

The most effective therapy to prevent bleeds among people with HA (PwHA) is the regular use (prophylaxis) of FVIII replacement, although episodic infusions may still be required to treat breakthrough bleeds (episodic treatment).^2^ However, some PwHA develop FVIII inhibitors, which are alloantibodies that neutralize the clotting activity of FVIII.^2,3^ This occurs in approximately 20%–30% of severe and 5%–10% of moderate/mild PwHA.^1^ Consequently, as PwHA and inhibitors (PwHAi) present higher mortality and morbidity than PwHA without inhibitors,^4,5^ they experience decreased social and emotional functioning, physical pain/discomfort, and arthropathy.^6^

To revert these outcomes, immune tolerance induction (ITI) is indicated.^7^ ITI comprises the administration of repeated doses of FVIII to eradicate inhibitors.^7^ Nonetheless, this treatment is unsuccessful in approximately 30%–40% of PwHAi, who will ultimately require bypassing agents (BPA) for both prophylactic and episodic treatments.^7^ The current available BPA are activated prothrombin complex concentrate (aPCC) and recombinant activated FVII (rFVIIa).^8^ They have similar effectiveness as prophylactic or episodic therapeutics.^9^

However, this scenario has changed since the advent of emicizumab, a humanized, bispecific monoclonal antibody. Emicizumab acts as a FVIII-mimetic agent, linking to the activated FIX (FIXa) and FX to reestablish the coagulation process.^10^ In addition, since the structure of emicizumab has no homology with FVIII, it is not neutralized by the anti-FVIII inhibitors.^11^ Finally, compared with BPA prophylaxis, emicizumab prophylaxis has demonstrated promising results in some publications.^12-15^ Further potential benefits are its bioavailability after subcutaneous administration and its increased half-life, which demands weekly or even monthly infusions.^10^ Despite the apparent advantages, no systematic review has analyzed its actual benefits as prophylaxis for PwHAi compared with BPA prophylaxis.

## OBJECTIVE

We conducted a systematic review and meta-analysis to compare bleeding endpoints between PwHAi on emicizumab and those on BPA prophylaxis.

## METHODS

### Protocol and registration

This systematic review was registered in the International Prospective Register of Systematic Reviews (PROSPERO; CRD42021278726). We conducted the systematic review according to the Cochrane recommendations^16^ and reported it according to the Preferred Reporting Items for Systematic Reviews and Meta-Analyses (PRISMA) statement (Supplementary Table 1).^17^ The research question was “Is emicizumab prophylaxis effective at reducing the bleeding endpoints among PwHAi when compared with BPA prophylaxis?”.

**Table 1:** Search strategies in each platform (February 21, 2023)

Platform	Search strategy	Number of publications
CENTRAL	(((((hemophilia) OR (haemophilia)) OR (factor VIII)) OR (FVIII)) AND (((inhibitor) OR (anti-factor VIII)) OR (anti-FVIII))) AND (((emicizumab) OR (ACE910)) OR (hemlibra))	30
MEDLINE	(“hemophilia A”) OR (“haemophilia A”) AND (emicizumab OR hemlibra OR ACE910) AND (inhibitor OR anti-FVIII OR anti-factor VIII)	318
LILACS	“hemophilia A” OR “haemophilia A” [Words] and emicizumab OR hemlibra OR ACE910 [Words] and inhibitor OR anti-FVIII OR anti-factor VIII [Words]	01
Scopus	( ( hemophilia OR haemophilia OR factor AND viii OR fviii ) AND ( inhibitor OR “anti-factor viii” OR “anti-fviii” ) AND ( emicizumab OR ace910 OR hemlibra ) ) AND ( LIMIT-TO ( DOCTYPE , “ar” ) )	194

Table legend: CENTRAL = Cochrane Central Register of Controlled Trials; MEDLINE = Medical Literature Analysis and Retrieval System Online; LILACS = Literatura Latino-Americana e do Caribe em Ciências da Saúde. CENTRAL = Cochrane Central Register of Controlled Trials; MEDLINE = Medical Literature Analysis and Retrieval System Online; LILACS = Literatura Latino-Americana e do Caribe em Ciências da Saúde.

### Literature search

A literature search was performed by two authors on February 21, 2023. Specific search strategies were used for each of the following databases: Cochrane Central Register of Controlled Trials (CENTRAL); Medical Literature Analysis and Retrieval System Online (MEDLINE) via PubMed; Literatura Latino-Americana e do Caribe em Ciências da Saúde (LILACS; in English, Latin-American, and Caribbean Center on Health Sciences Information); and Scopus (Table 1). In addition, we manually searched the reference lists of published reviews retrieved from MEDLINE to obtain additional publications that met the eligibility criteria. We also accessed the ClinicalTrials platform (www.clinicaltrials.gov) on February 21, 2023, using “hemophilia A with inhibitor” in the “condition or disease” section and “emicizumab” in the “other terms” section, to detect registered studies and used their identification numbers to search for potential missing publications.

### Inclusion and exclusion criteria

The included publications presented information on the bleeding endpoints among PwHAi on emicizumab prophylaxis compared to those on BPA prophylaxis. Randomized and nonrandomized controlled trials, as well as observational studies were included. No restrictions on the publication date or language were applied. Publications were excluded for the following reasons: absence of bleeding evaluation; lack of data on emicizumab prophylaxis; publication type other than original article (for example, reviews or posters); absence of discrimination between data on PwHAi and PwHA without inhibitors; existence of a more recent publication with the same population; or absence of PwHAi on BPA prophylaxis.

### Publication selection

The web-based app Rayyan (https://www.rayyan.ai/) was used in the screening process.^18^ After the exclusion of duplicates, titles and abstracts were independently screened according to the inclusion criteria by two authors. Subsequently, publications that potentially fit the inclusion criteria were read entirely by the two authors to decide on inclusion in the systematic review. Discussions on contrasting selection results were conducted by the coordinators. We contacted the authors of publications that did not contain the information required for inclusion, specifically requesting lacking data. If such data were not provided, the publication was excluded.

### Data extraction

Data were extracted from the publications selected for the systematic review by two authors using a standardized form. This file contains information on the authors of the publication, study design, country(ies) where the studies were conducted, population size, population characteristics (age, sex, disease severity, and inhibitor titer), emicizumab/BPA regimens, and adverse events (AEs). Moreover, we collected information on bleeding endpoints as the main outcome of this systematic review.

### Risk of bias assessment

The risk of bias was assessed by two authors using the Joanna Briggs Institute (JBI) critical appraisal checklists for quasi-experimental trials, randomized controlled trials, and cohort studies.^19^ Following the JBI guidelines, we did not define cut-off values for categorizing the publications as having low, moderate, or high risk of bias^19^. Conversely, we presented the overall risk of bias for each domain.

Publication bias was not assessed owing to the small number of publications included in this systematic review.

### Meta-analysis

The main outcome analyzed was the annualized bleeding rate (ABR) for treated bleeds. Initially, we calculated the mean and standard deviation (SD) of the study samples based on the median and range according to the methodology described by Hozo et al.^20^ Heterogeneity between studies was evaluated using the *I* ^2^ statistic. *I* ^2^ values of 60%–100%, 40%–59%, and 0%–39% indicated high, moderate, and low heterogeneity, respectively.^21^ We also used the Cochran’s *Q* test was used to verify the heterogeneity between the selected studies.^22^ The null hypothesis was that all studies were identical. Next, random- or fixed-effects models were used to analyze the magnitude of effect on ABR for treated bleeds after intervention implementation, when study heterogeneity was high (≥ 50%). A meta-analysis was conducted for all pooled studies that evaluated the emicizumab maintenance regimen of 1.5 mg/kg weekly according to the type of study (controlled trials and cohort) as subgroups. Statistical inference was performed using the Student’s *t* test. The effect size was presented as the standardized mean difference for the pre-post studies with the respective 95% confidence interval (95%CI). Forest plots were generated to visualize the results. Analyses were conducted using the R software version 4.1.3 (R Core Team, Vienna, Austria).^23^

## RESULTS

### Publication selection

**Figure 1:**
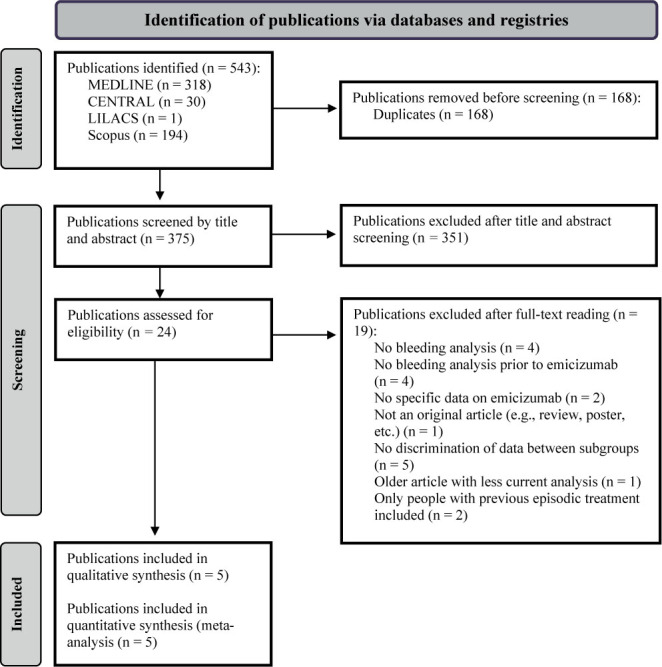
The Preferred Reporting Items for Systematic reviews and Meta-Analyses (PRISMA) flowchart for study selection.

The literature search retrieved 543 publications. After duplicate removal and title/abstract screening, 24 publications were evaluated (Supplementary Table 2). Subsequently, 19 publications were excluded because they did not completely fulfill the inclusion criteria. Thus, five publications were included in the final version: one randomized controlled trial,^12^ two non-randomized controlled trials,^13,24^ and two cohort studies.^25,26^ A PRISMA diagram of the selection process is presented in Figure 1. A search of the reference lists did not yield any new publications.

**Table 2:** Characteristics of the populations in the included publications

First author, year	Study design	Sponsor	Country (ies)	N	Age	Sex	HA severity	Inhibitor titer (BU/mL)	BPA prophylaxis regimen	Emicizumab prophylaxis regimen: loading dose	Emicizumab prophylaxis regimen: maintenance dose	ABR for treated bleeds during BPA prophylaxis	ABR for treated bleeds during emicizumab prophylaxis	Adverse events*
Oldenburg, 2017^12^	Controlled RCT	Roche and Chugai Pharmaceutical	Spain, Costa Rica, USA, Italy, United Kingdom, Germany, Japan, Poland, Australia, Republic of Korea, France, South Africa, New Zealand, Taiwan	24	17#	M	NR	NR	NR	3.0 mg/kg QW × 4	1.5 mg/kg QW	Med: 11.5 Mean: 17.1 SD: 13.2	Med: 0 Mean: 9.7 SD: 11.1	Yes#
Young, 2019^13^	Controlled NRCT	Roche and Chugai Pharmaceutical	United Kingdom, USA, Spain, Germany, Italy, South Africa, Japan, Turkey, France, Costa Rica	15	<12	M	NR	>5	NR	3.0 mg/kg QW × 4	1.5 mg/kg QW	Med: 17.9 Mean: 20.4 SD: 13.3	Med: 0 Mean: 0.3 SD: 0.4	No 2 neutralizing ADA
Shima, 2021^24^	Controlled NRCT	Chugai Pharmaceutical	Japan	3	>12	M	Severe	NR	NR	3.0 mg/kg QW × 4	3.0 mg/kg QW	Med: 24.3 Mean: 25.4 SD: 3.5	Med: 0 Mean: 0.6 SD: 0.6	No
Misgav, 2021^25^	Cohort	None	Israel	2	62.9 (mean)	NR	Severe	35.6	>2 times/week	3.0 mg/kg QW × 4	1.5 mg/kg QW	Med: 15.5, Mean: 15.5 SD: 6.1	Med: 2.2 Mean: 2.2 SD: 0.6	No
McCary, 2020^26^	Cohort	None	USA	12	8.05 (median)	M	Severe	NR	4 times/week	3.0 mg/kg QW × 4	Not informed	Med: 2 Mean: 7.3 SD: 7.3	Med: 0 Mean: 0 SD: 0.2	No

*Related to emicizumab only;

# People with hemophilia A without and with inhibitors; ABR = annualized bleeding rate; ADA = anti-drug antibodies; BPA = bypassing agents; BU = Bethesda units; HA = hemophilia A; QW = weekly; M = male; Med = median; NR = not reported/not possible to be inferred; NRCT = non-randomized controlled trial; RCT = randomized controlled trial; SD = standard deviation; USA = United States of America.

CENTRAL = Cochrane Central Register of Controlled Trials; MEDLINE = Medical Literature Analysis and Retrieval System Online; LILACS = Literatura Latino-Americana e do Caribe em Ciências da Saúde.

In the ClinicalTrials database, 20 registered studies were identified. Among these, we excluded two trials that did not use emicizumab as the main investigated drug, four trials that did not include PwHAi cases, and one trial that had been revised to include two protocols. The analysis of the remaining 12 studies did not identify any new publications.

### Study characteristics

Among the selected publications, two controlled trials presented limited information regarding the specific subgroup of PwHAi that received previous prophylactic BPA (Table 2).^12,13^ However, the data of PwHAi who received episodic and prophylactic BPA are mixed in the tables and comparisons. Both studies included only some of the participants in the intra-individual analysis of ABR, thus presenting partial results of emicizumab prophylaxis *versus* BPA prophylaxis. In Shima et al.,^24^ Misgav et al.,^25^ and McCary et al.,^26^ comparisons involved only PwHAi on prophylaxis.

Overall, data of 56 European, Asian, and Central American male PwHAi were analyzed. We could not characterize the overall age range, HA severity, or inhibitor titer because some publications did not discriminate the data.

### Treatment regimens

The treatment regimens with emicizumab in the included publications consisted of loading doses of 3.0 mg/kg weekly for a month.^12,13,24-26^ The maintenance regimens consisted of weekly 1.5 mg/kg injections in most of the publications.^12,13,24,25^ McCary et al.^26^ did not specifically describe this information, mentioning that the regimens were either weekly, every 2 weeks, or every 4 weeks. The BPA prophylaxis regimen before study entry was partially detailed in two publications, in which 14 PwHAi received prophylactic BPA four times/week,^26^ or at least two times/week.^25^

### Risk of bias assessment

The evaluation of the risk of bias is described in Supplementary Figure 1. In two controlled trials, PwHAi on episodic treatment with BPA were evaluated together with PwHAi on BPA prophylaxis.^12,13^ In addition, in the study by Oldenburg et al.,^12^ the randomization method was not explained, and the assessors were not blinded. None of the publications adjusted the outcomes for potential confounding factors, such as target joints and disease severity.^12,13,24-26^

### Bleeding endpoints

Several methods have been used for bleed evaluation^12,13^ (Supplementary Table 3). ABR for treated bleeds was the only common method used in all the publications (Table 2). However, while the randomized controlled trial, one non-randomized controlled trial, and cohort studies performed inferential statistics,^12,13,25,26^ only a descriptive analysis was performed by Shima et al.^24^

Oldenburg et al.^12^ have reported that 80% of PwHAi on emicizumab prophylaxis experienced a reduced median ABR for treated bleeds compared to those on BPA prophylaxis. Young et al.^13^ described that emicizumab prophylaxis prevented more bleeding than BPA prophylaxis. Shima et al.^24^ have reported a higher efficacy of emicizumab prophylaxis than BPA prophylaxis, with the median ABR for treated bleeds reduced to zero. Additionally, all PwHAi in cohort studies experienced reduced bleeding rates.^25,26^

### ABR for treated bleeds

For the subgroup of PwHAi analyzed by Oldenburg et al.,^12^ the median ABR for treated bleeds was 11.5, during BPA prophylaxis and decreased to zero during emicizumab prophylaxis. In a study by Young et al.,^13^ the ABR for treated bleed was reduced from 17.9 during BPA prophylaxis to zero during emicizumab prophylaxis. In addition, while the overall median ABR for treated bleeds in Shima et al.^24^ was 24.3 among PwHAi on BPA prophylaxis, it decreased to zero during emicizumab prophylaxis. Lastly, in cohort studies,^25,26^ the median ABR for treated bleeds was reduced from 2 and 15.5 during BPA prophylaxis to 0 and 2.2 during emicizumab prophylaxis, respectively.

**Figure 2:**
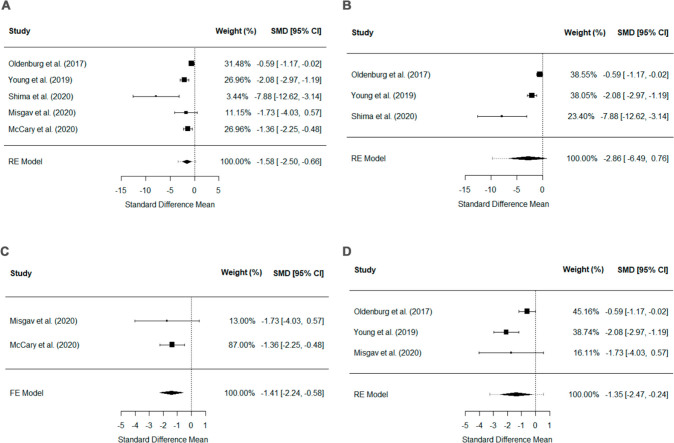
Forest plot comparing the annualized bleeding rate for treated bleeds among people with hemophilia A and inhibitors on emicizumab prophylaxis versus bypassing agents prophylaxis in A) all the included publications; B) publications that used the 1.5 mg/kg weekly regimen for emicizumab prophylaxis; C) cohort studies; and D) controlled trials. FE = fixed effect; RE = randomized effect; SMD = standard mean deviation; 95%CI = 95% confidence interval.

The results of the meta-analysis are presented in Figure 2. Altogether, all publications^12,13,24–26^ reported decreased ABR for treated bleeds with emicizumab prophylaxis compared to those treated with BPA prophylaxis (P = 0.0008), with severe heterogeneity (*I*² = 68.4%) (Figure 2A). A decrease in ABR for treated bleeds with emicizumab prophylaxis in comparison with those treated with BPA prophylaxis was also observed when only the PwHAi on the 1.5 mg/kg weekly emicizumab regimen was analyzed (P = 0.0173),^12,13,25^ with severe heterogeneity (*I*² = 71,4%) (Figure 2B), and when only the cohort studies were evaluated (P = 0.0008),^25,26^ with mild heterogeneity (*I*² = 0.0%) (Figure 2C). Regarding the separate analysis of controlled trials,^12,13,24^ no difference in the ABR for treated bleeds between the treatments was noted (P = 0.1220) (Figure 2D).

### Safety

All the publications reported on safety issues regarding emicizumab prophylaxis.^12,13,24-26^ In one trial, five reported thrombotic events associated with the concomitant use of emicizumab and high daily doses of aPCC for > 1 day.^12^ All the cases resolved after aPCC was interrupted, and two participants resumed emicizumab prophylaxis. Despite the resolution, one participant died because of bleeding. No other thrombotic events have been reported in other publications.^13,24-26^

Only one publication reported the development of neutralizing anti-drug antibodies (ADA) in two PwHAi,^13^ one of which presented a loss of efficacy, leading to discontinuation. The other individual remained in the trial because his neutralizing ADA levels were undetectable after 2 months. Moreover, although no participant in the study by Oldenburg et al.^12^ tested positive for ADA, two of them presented with decreased emicizumab plasma concentrations over time. However, no increase in bleeding was observed until the end of the study. Other AEs, such as nasopharyngitis, infection-site reactions, headaches, and rhabdomyolysis, were either mild, moderate, or deemed unrelated to emicizumab.^12,13,24-26^

## DISCUSSION

Meta-analysis of the pooled data from this systematic review confirmed that emicizumab prophylaxis is superior to BPA prophylaxis in reducing the ABR for treated bleeds in PwHAi. Interestingly, meta-analysis of controlled trials sponsored by pharmaceutical industries did not indicate differences between emicizumab and BPA as prophylaxis for PwHAi. Differences were only detected when cohort studies were included in the pooled data.

Several publications were not included in this review because we could not separate results from PwHA without and with inhibitors, and those receiving episodic and prophylactic treatments with BPA.^27,28^ They have demonstrated that emicizumab prophylaxis indeed reduced bleeds in comparison to both previous prophylactic and episodic treatment with factors.^27,28^ In an Israeli publication, ABR for treated bleeds decreased from 2 (0–30) during BPA treatment to 1 (0–3) in PwHAi on emicizumab prophylaxis compared with previous prophylactic and episodic treatments.^27^ In addition, although Barg et al.^28^ did not analyze ABR, they have reported that almost 65% of PwHAi did not need additional hemostatic treatment other than emicizumab.

However, some publications still present conflicting information, demonstrating a variable response to emicizumab prophylaxis and a considerable persistent amount of breakthrough bleeds.^29,30^ In a prospective study, half of the patients, including PwHAi, still had bleeds while on emicizumab prophylaxis.^29^ In addition, Warren et al.^30^ described a wide variability in bleeding rates in PwHAi on emicizumab prophylaxis, ranging from 0 to 6.1, although the majority of events were related to trauma.

Conflicts are not only related to bleeding but also to the safety of emicizumab. Thrombotic events, some of which are fatal, have been reported in three publications.^12,29,31^ The HAVEN 1 controlled trial detected these events in association with the use of aPCC, including three thrombotic microangiopathies (TMA), which led researchers to change treatment protocols for breakthrough bleeds.^12^ Since then, no more events have been reported in controlled trials. By contrast, in real-world evaluations, other thrombotic events continue to be detected, one of them associated with the use of aPCC 30 days after emicizumab was discontinued.^29,31^ This may be a consequence of the persistence of emicizumab in the blood for approximately 6 months.^32^ Postmarketing evaluations have also revealed venous thrombosis and one additional case of TMA in PwHA, although inhibitor status was not provided.^33^ Moreover, one PwHAi on emicizumab prophylaxis developed myocardial infarction 36 h after administered with rFVIIa for a bleeding episode.^34^ These situations expose the need for continuous monitoring of thrombotic events in order to clarify their relationship with this new drug.

Another safety issue involves the development of neutralizing ADA. They were first detected in two PwHAi enrolled in a publication that included this systematic review.^12,13^ A case report of a pediatric PwHAi has also been published, resulting in emicizumab discontinuation.^35^ Furthermore, the development of non-neutralizing ADA was also noted in 11 PwHAi of the HAVEN trials.^36^ This is an important issue because in case emicizumab no longer prevents bleeds, BPA prophylaxis needs to be resumed.^7^

One important aspect of the meta-analysis results was the high heterogeneity among publications. This finding may be attributed to differences in study designs,^37^ variations in intervention methods (dosage and duration),^37^ and publication bias, which could not be assessed.^38^

This systematic review had some limitations. First, the small number of publications included revealed that few studies were conducted to allow for a more thorough evaluation of PwHAi. In addition, the number of PwHAi who received BPA prophylaxis and then switched to emicizumab prophylaxis was small. Second, the fact that not all publications discriminated data based on inhibitor status and prior BPA prophylaxis limited the potential inclusion of comparisons in the meta-analysis. The meta-analysis also presents limitations. Because of the small number of publications, we merged data from controlled trials and cohort studies to integrate the results, which may influence the interpretability of the findings considering the heterogeneous study designs and quality of evidence. Additionally, the different dosages, age ranges, and small populations across studies warrant careful evaluation of the results. Finally, the difficulty regarding the analysis of AEs was also an issue, as we were unable to identify which events occurred specifically in the population of PwHAi under BPA prophylaxis who switched to emicizumab prophylaxis.

PwHAi have more bleeds and more difficult-to-treat bleeds than their non-inhibitor counterparts.^39,40^ Hence, morbidity, including hemophilic arthropathy and worse quality of life, and mortality secondary to hemorrhage are more frequent among PwHAi than those PwHA without inhibitors.^4,5,41^ The BPA prophylaxis has an effectiveness of approximately 60%–72%,^42,43^ implying that bleeding events may still occur.^44^ In addition, up to 20% of the bleeding events in PwHAi may not be controlled with any BPA on usual recommended regimens.^40,45^ Therefore, the reduction of ABR due to BPA prophylaxis for PwHAi may not be followed by significant joint health and quality of life improvements and reduced mortality compared with those PwHAi exclusively treated on-demand.^43,44^ The introduction of more effective prophylactic therapeutic (i.e., emicizumab) in the armamentarium for treating PwHAi may result in better avoidance of hemophilic arthropathy,^46,47^ assurance of an adequate quality of life,^48,49^ and maintenance of mortality similar to the people without hemophilia. Hence, future research should focus on separate analyses of PwHAi from those without inhibitors, specially evaluating concurrent (head-to-head) emicizumab and BPA prophylaxes in a pre-calculated population size, as well as a better description of prior BPA prophylaxis and AEs.

## CONCLUSION

This systematic review and meta-analysis demonstrated that emicizumab prophylaxis was superior to BPA prophylaxis in preventing ABR for treated bleeds in PwHAi. However, the results should be interpreted with caution because of the small population size and potential risk of bias.
